# A centenary tale: population genetic insights into the introduction history of the oriental fire-bellied toad (*Bombina orientalis*) in Beijing

**DOI:** 10.1186/s12862-022-02072-z

**Published:** 2022-10-14

**Authors:** Shan Zhang, Meixi Lin, Jiawei Liu, Jiangce Chen, Dong Liu, Jindong Zhao, Meng Yao

**Affiliations:** 1 School of Life Sciences, Peking University, R312, School of Life Sciences Bldg., 100871 Beijing, China; 2 Institute of Ecology, College of Urban and Environmental Sciences, Peking University, Beijing, 100871 China; 3 Department of Ecology and Evolutionary Biology, University of California-Los Angeles, Los Angeles, CA 90095 USA; 4 Department of Ecology and Evolution, University of Chicago, Chicago, IL 60637 USA; 5 Mechanical Engineering Department, University of Connecticut, Storrs, CT 06269 USA; 6 Department of Biology, School of Life Sciences, Southern University of Science and Technology, Shenzhen, 518055 China

**Keywords:** Amphibian, Founder effect, Founder size, Genetic bottleneck, Introduction success, Propagule pressure

## Abstract

**Background:**

The successful establishment of a species population following a single introduction of a few individuals to a non-native area has been limited. Nevertheless, the oriental fire-bellied toad (*Bombina orientalis*) population in Beijing is purportedly descended from a single introduction of about 200 individuals translocated from Yantai, Shandong Province, China, in 1927.

**Results:**

To resolve the introduction process and to understand the genetic consequences since that introduction approximately 90 years ago, we investigated the population’s genetic diversity and structure using 261 toads from Beijing and two native Shandong populations and inferred the species’ introduction history using simulation-based approaches. Analysis of mitochondrial DNA (mtDNA) sequences showed the two haplotypes found in Beijing nested within Yantai haplotypes, thus corroborating the historical record of the translocation source. The mtDNA and 11 nuclear microsatellite markers revealed both considerably lower genetic diversity in Beijing than in the source population and strong genetic differentiation between them. Although the current census population in Beijing may be in the range of a few thousand, the effective population size was estimated at only 20–57. Simulations also suggest that this population may have descended from 40–60 founders.

**Conclusions:**

The Beijing population’s genetic patterns were consistent with the consequences of a severe bottleneck during introduction followed by genetic drift. The introduction trajectory constructed for this *B. orientalis* population reveals the genetic footprints of a small population sustained in isolation for nearly a century. Our results provide an intriguing example of establishment success from limited founders and may inform ex situ conservation efforts as well as the management of biological invasions.

**Supplementary Information:**

The online version contains supplementary material available at 10.1186/s12862-022-02072-z.

## Background

Genetic diversity is essential for maintaining the viability and adaptive potential of wild populations [1, 2]. Small, isolated populations are at a higher risk of extinction than are large, connected ones. That is due mainly to the low genetic diversity and hampered fitness and adaptability that result from genetic drift and inbreeding, as well as to the high tendency to succumb to stochastic events [3, 4]. Therefore, for a species to successfully establish in a non-native range, it is expected to satisfy an introduction effort (i.e., the number of individuals introduced and the frequency of introductions), also referred to as ‘propagule pressure’ [5, 6]. Indeed, both field observations and laboratory experiments have shown that larger founding populations or multiple introductions typically convey greater establishment probabilities than do small founding populations and single introductions [7–11].

However, some species have successfully, although rarely, established populations from a single introduction of a few founders, and those populations have persisted in isolation from other populations [11–13]. Such introduced populations are expected to have very low genetic diversity levels because initial founder bottlenecks allow only limited gene pools to propagate, and subsequently the strong genetic drift causes random allele loss in small populations [14–17]. Such populations, if substantiated, seriously challenge the widely accepted hypothesis that genetic variation is essential for both species survival and continued population persistence. Understanding the genetic and demographic processes of the introduction and propagation of such populations would provide important insights for managing biological invasions and can be used to inform ex situ conservation and re-introduction of locally extirpated species [18, 19].

A major drawback of many studies of introduction genetics lies in the short time frame relative to the life history of the study species, especially vertebrates. Most non-native animal populations with known introduction histories have established within the last few decades, and have produced only a few generations (e.g., 4–10 generations in the introduced populations of the golden lion tamarin *Leontopithecus rosalia* [20], hihi *Notiomystis cincta* [19], and Alpine ibex *Capra ibex* [21]). Those short introduction periods may not allow genetic effects to fully manifest. That, in turn, precludes investigations of the long-term consequences of persistence in isolated populations, and that hinders both the understanding of population genetic dynamics and the assessment of introduction success in longer time frames.

**Fig. 1:**
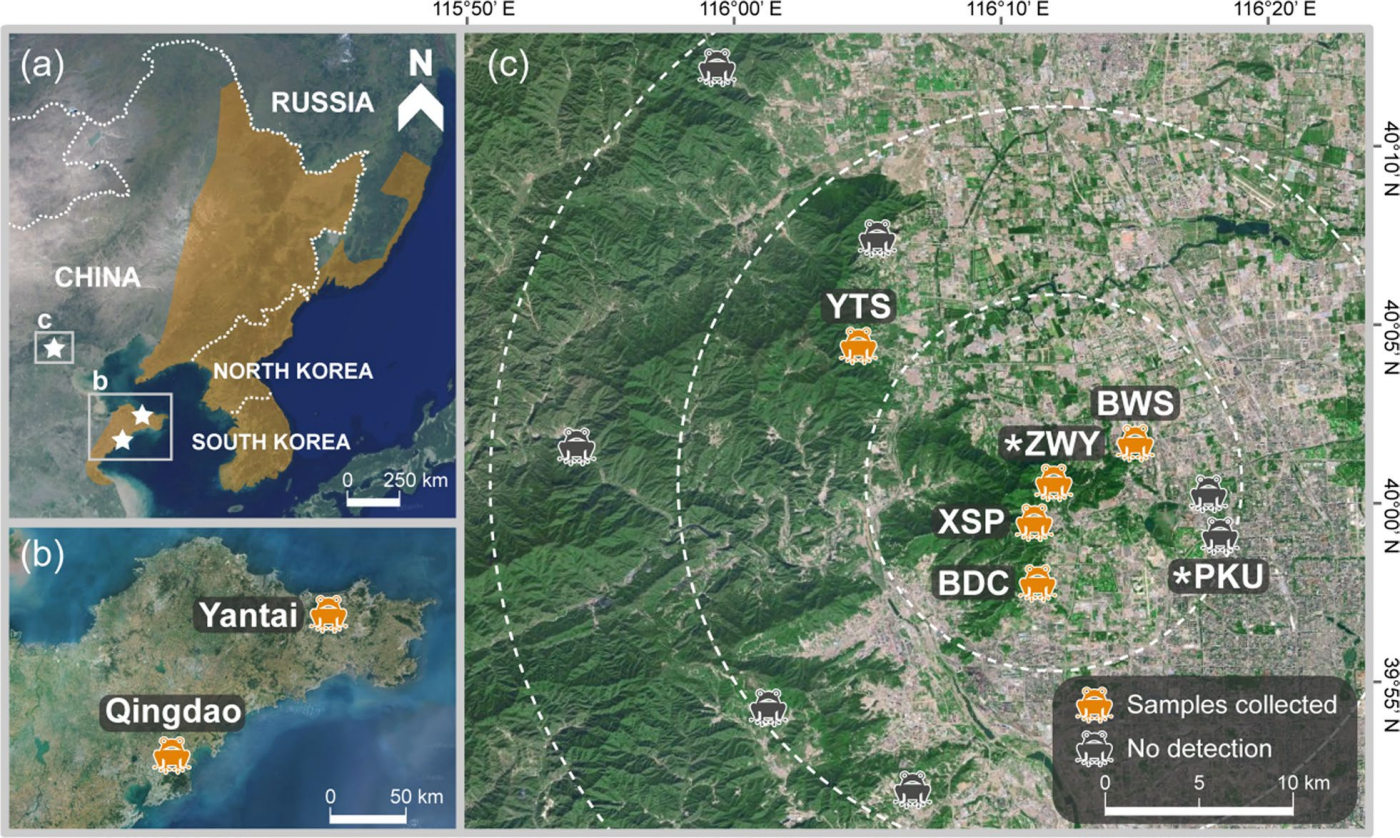
*Bombina orientalis* ranges and sampling sites. **a** The species natural distribution range shown in brown (Source: the IUCN Red List of Threatened Species, version 2021–2). White stars indicate the sampling areas. **b** Sampling sites in Shandong Province (Shandong Peninsula). **c** Sampling sites in Beijing. PKU, Peking University; ZWY, Zhiwuyuan; XSP, Xiangshan Park; BWS, Baiwangshan; YTS, Yangtaishan; BDC, Badachu. Orange ‘toad’ icons show survey sites where individuals were found and dark gray icons indicate survey sites with no detected individuals. Asterisks indicate the original introduction sites. Dotted circles represent 10, 20, and 30 km distances from the ZWY initial introduction site

The oriental fire-bellied toad, *Bombina orientalis*, is a small semiaquatic species found mostly in two discontinuous ranges: one in the Korean peninsula and adjacent regions of northeastern China and the Russian Far East and the other in a smaller range in the Shandong Peninsula of China (Fig. 1a, b). Although the species is currently considered of Least Concern by the IUCN [22], its range-wide population is decreasing, due mainly to habitat loss and degradation [23]. Beijing, though located within the altitudinal zone of the species’ natural ranges, is not part of the species’ native area. In 1927, the Chinese herpetologist Chengzhao Liu (1900–1976) moved about 200 *B. orientalis* toads from Yantai City in Shandong Province to Beijing and released them in ditches on the Yenching University campus (now Peking University [PKU] campus) and in a stream near the Wofo Temple (now in Zhiwuyuan Park [ZWY]) [24] (Fig. 1c). The purpose of this translocation was undocumented and the toads were not systematically monitored afterwards, so little is known about their population demographic dynamics after their introduction. Several decades later, Liu and Hu [24] found no *B. orientalis* at the PKU site, but did find them at ZWY. With no known further introduction efforts and a lack of genetic exchange with other populations, the currently surviving Beijing population represents a case of successful establishment from a single introduction of a limited number of individuals. That has provided a rare opportunity to gain knowledge of the demographic and genetic consequences that befell a small, isolated vertebrate population over a time frame of approximately 90 years, or 30 generations (assuming a generation time of three years [25]).

The goal of the present study was to reconstruct the introduction history and the population genetic trajectory of *B. orientalis* in Beijing. We conducted field surveys of this species in Beijing (Fig. 1c) and used both mitochondrial DNA (mtDNA) and nuclear microsatellite markers to quantify that population’s genetic diversity, subsequently comparing it with its alleged source population in Yantai, as well as with a non-source native population in Qingdao City, Shandong Province (Fig. 1b). Specifically, we assessed the effects of introduction and establishment on the population genetic variation of the Beijing population in relation to that of native populations. Also, by simulating genetic processes under possible demographic scenarios, we reconstructed the Beijing population’s introduction history and estimated the number of its founders.

## Results

### Field surveys and sampling

We detected *B. orientalis* at five (ZWY, XSP, BDC, BWS, and YTS) of the 12 sites that we surveyed in Beijing during 2015–2018, and we collected 13–49 toe clip samples from toads at each site, for a total of 150 samples (Fig. 1c; Table 1; Additional file 1: Table S1). Toads were found mostly in and near mountain streams or small ponds in foothill areas. The sampled sites were all located within 13 km of the ZWY release site. The species was not found at the other release site, PKU. Based on a one-time mark-recapture experiment at the XSP site, we calculated 197 toads visiting a single waterhole. Extrapolating from that, our rough estimate for the total Beijing population size (*N*_c_) was about 1,500–4,000 (see “Methods” section).

**Table 1:** Summary of *Bombina orientalis* genetic diversity parameters estimated using the mtDNA and microsatellite loci of different populations

Population	*N*	mtDNA				*N*_A_	Microsatellites						
		*n*	*h* (SD)	*π* (SD)	Unique Haplotypes		*N*_PA_	*N*_a_	*A*_R_	*A*_P_	*H*_O_	*H*_E_	*F*_IS_
All Beijing	150	2	0.433 (0.029)	0.0014 (0.0000)	1	72	8	6.0	4.07	1.13	0.521	0.581	0.104
ZWY	49	2	0.499 (0.048)	0.0015 (0.0000)	0	64	1	5.3	4.19	0.17	0.550	0.602	0.087
XSP	51	2	0.500 (0.023)	0.0016 (0.0000)	0	56	0	4.7	3.88	0.08	0.534	0.550	0.031
BWS	22	2	0.506 (0.050)	0.0017 (0.0000)	0	47	0	3.9	3.40	0.04	0.509	0.483	–0.055
YTS	15	1	0.000 (0.000)	0.0000 (0.0000)	0	34	0	2.8	2.72	0.07	0.433	0.416	–0.043
BDC	13	1	0.000 (0.000)	0.0000 (0.0000)	0	40	1	3.3	3.26	0.12	0.468	0.459	–0.022
Yantai	89	38	0.967 (0.007)	0.0029 (0.0000)	37	207	88	17.3	9.21	3.87	0.700	0.795	0.120
Qingdao	22	10	0.905 (0.035)	0.0031 (0.0000)	10	116	21	9.7	7.75	3.04	0.668	0.778	0.145

For mtDNA (combined sequences of the Cytb and D-loop region; ~ 1830 bp) data: *N*, sample size; *n*, number of haplotypes; *h*, haplotype diversity; *π*, nucleotide diversity; SD, standard deviation. For microsatellite data: *N*_A_, the total number of alleles; *N*_PA_, the number of private alleles; *N*_a_, mean number of alleles per locus; *A*_R_, allelic richness; *A*_P_, private allelic richness; *H*_O_, observed heterozygosity; *H*_E_, expected heterozygosity; *F*_IS_, inbreeding coefficient. Unique haplotypes and private allele parameters were first assessed for three local populations (i.e., all Beijing, Yantai, and Qingdao), followed by the assessment of Yantai, Qingdao, and each of the Beijing sites separately (i.e., seven populations). ZWY, Zhiwuyuan; XSP, Xiangshan Park; BWS, Baiwangshan; YTS, Yangtaishan; BDC, Badachu

We also collected 89 and 22 toad samples in Yantai and Qingdao, respectively, for comparisons between the introduced (Beijing) and the alleged source population (Yantai) and a non-source native population (Qingdao) (Fig. 1b).

### Genetic diversity

**Fig. 2:**
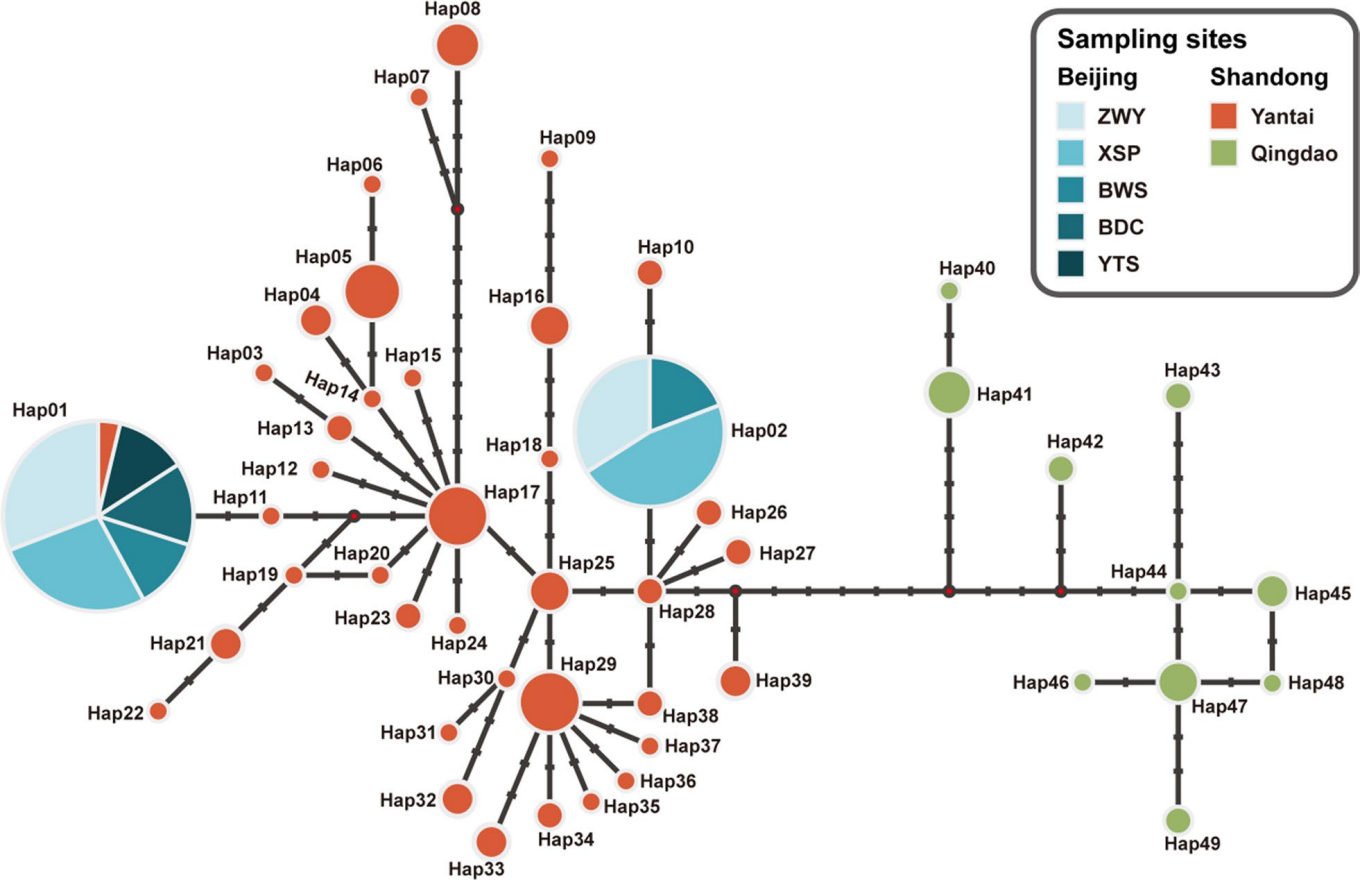
The median-joining haplotype network of the combined mtDNA sequences. Each circle represents a unique haplotype. Circle size indicates the number of individuals (varying between 1 and 107) and colors reflect different sampling sites. The transverse dashes on the lines indicate substitutions between haplotypes. See Fig. 1 for sampling site information

We successfully obtained the Cytb and D-loop sequences from all 261 samples. We found 38 and 10 haplotypes of the combined mtDNA sequences in the Yantai and Qingdao samples, respectively, but only two unique haplotypes in the 150 Beijing samples (Additional file 1: Table S2), thus demonstrating a remarkably lower mtDNA diversity in the Beijing than in the two native populations (Table 1). Specifically, both the haplotype diversity and nucleotide diversity of all Beijing samples were less than half that of the Yantai and Qingdao samples. A median-joining network of the haplotypes showed that samples from Yantai and Qingdao formed two distinct haplogroups with at least a 12-nucleotide difference between haplotypes from each group (Fig. 2). The two haplotypes found in the Beijing samples (Hap01 and Hap02) were nested within the Yantai haplogroup, with one of the haplotypes (Hap01) also present in four Yantai samples. Hap02 was not found in Yantai samples, but the sequence only differed by one nucleotide from the two most similar haplotypes detected in Yantai.

Most samples were successfully genotyped at all microsatellite loci (229 samples having data at 12 loci, 31 at 11 loci, and 1 at 10 loci; primer information shown in Additional file 1: Table S3), so all samples were included in the genetic analysis. One microsatellite locus (I165) showed a departure from Hardy–Weinberg equilibrium in each of the three local populations (Beijing, Yantai, and Qingdao; Additional file 1: Table S4). Two loci (I165 and H102) showed signs of null alleles in each of the local populations. We detected no consistent linkage disequilibrium pattern between any pair of loci across populations. Therefore, we only removed locus I165 and retained the other 11 loci for subsequent analyses. Compared with the Yantai and Qingdao samples, the Beijing samples had a considerably reduced nuclear genetic diversity, as shown by its substantially smaller total number of alleles (*N*_A_), number of private alleles, allelic richness (*A*_R_), and observed (*H*_O_) and expected (*H*_E_) heterozygosities (Table 1). Specifically, the *N*_A_, *A*_R_, *H*_O_, and *H*_E_ in Beijing samples were all significantly lower than those in the Yantai population (*p* < 0.05 for all paired *t*-tests; Additional file 1: Table S5). However, the extent of inbreeding, as measured by the inbreeding coefficients, between the Beijing and Yantai populations did not differ significantly (Table 1 and Additional file 1: Table S5). Furthermore, when considered as a whole, the Beijing population contained eight private alleles that were not found in the Yantai and Qingdao samples, but the Beijing samples possessed no or only one private allele among the five sites (Table 1).

### Population differentiation

**Fig. 3:**
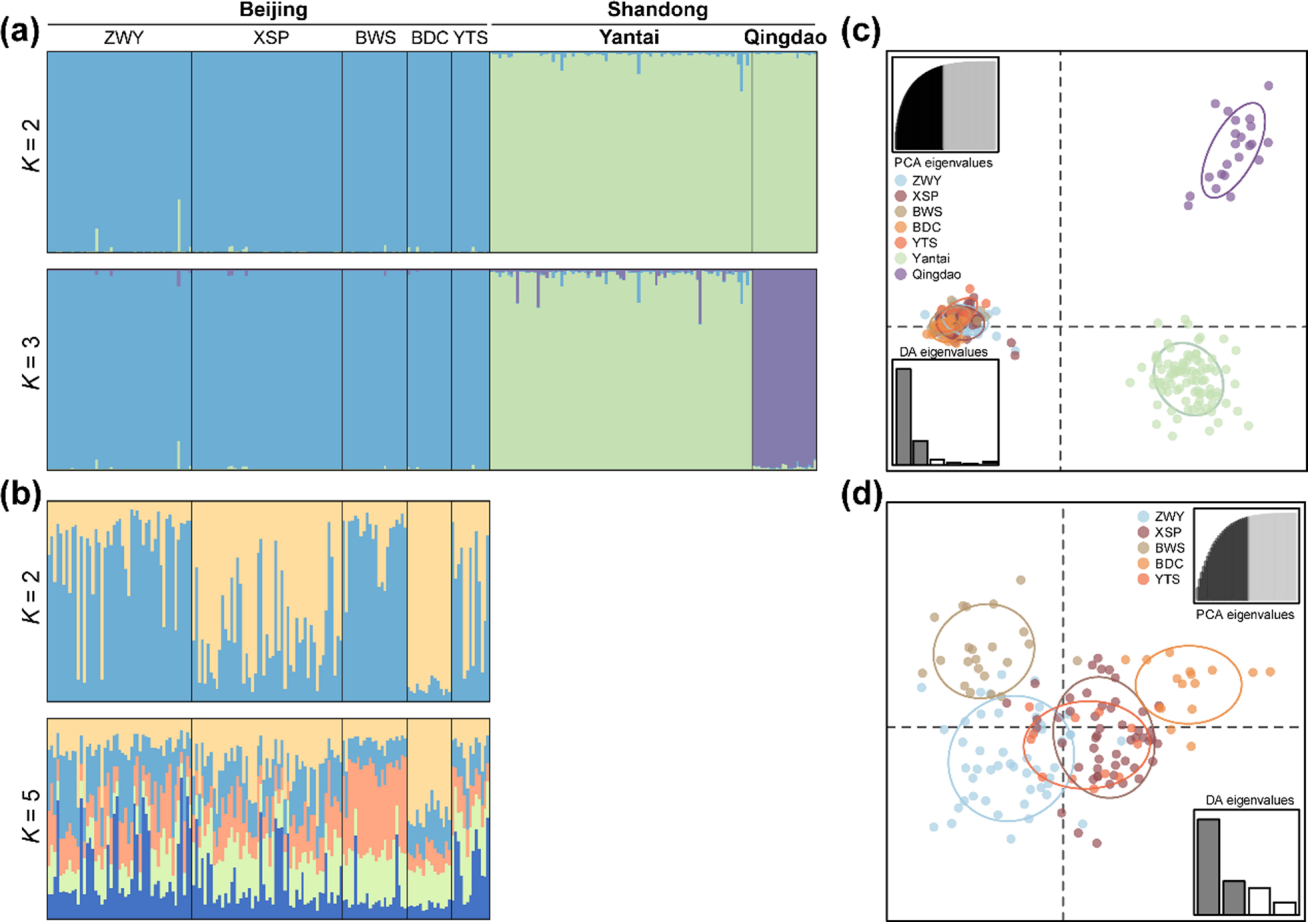
*Bombina orientalis* population differentiation analysis based on microsatellite data. Genetic clustering using STRUCTURE of (**a**) all samples and of (**b**) samples from Beijing only. The vertical lines are broken into different colors showing the proportion of each individual assigned to each of the inferred clusters. Letters at the top of the figure correspond to sampling locations. *K*, number of genetic clusters. Discriminant analysis of principle components plots of the genetic structure based on individual genotypes among samples from (**c**) all sampling sites and from (**d**) sites in Beijing. Dots represent individuals and inertia ellipses indicate defined groups by color. The top insets in each graph indicate the cumulative variance explained by retained PCA eigenvalues and the bottom insets represent linear discriminant analysis (DA) eigenvalues of the first few principal components. See Fig. 1 for sampling site information

We investigated the population genetic differentiation using the microsatellite data. First, the Bayesian clustering method implemented in STRUCTURE suggested that the number of genetic clusters (*K*) = 2, in which the Yantai and Qingdao samples clustered together and the Beijing samples clustered separately, was the best supported result (Additional file 1: Fig. S1a,c). Also, when *K* = 3 there was clear clustering that corresponded to the partitions between the Beijing, Yantai, and Qingdao samples (Fig. 3a). The three clusters showed distinct boundaries with no individual having apparent admixture, thus indicating strong genetic differentiation between populations. Subsequent STRUCTURE analyses on the Beijing samples alone did not detect further division at *K* = 2 through 5 (Fig. 3b; Additional file 1: Fig. S1b,d), thus suggesting a lack of genetic divergence within the Beijing population. Discriminant analysis of principle components (DAPC) population clustering results showed overall patterns consistent with the STRUCTURE analyses, in which the Beijing, Yantai, and Qingdao samples formed distinct clusters (Fig. 3c), and the samples from the Beijing sites had generally limited differentiation, except that the BWS and YTS samples were slightly separated from the others (Fig. 3d).

The Beijing samples showed strong genetic divergence from those of the Yantai and Qingdao populations, as measured by both pairwise *R*_ST_ based on microsatellite data and *Φ*_ST_ based on mtDNA data (all *p* < 0.001; Table 2). Also, pairwise comparison values were smaller for Beijing-Yantai than for Beijing-Qingdao.

**Table 2:** Pairwise genetic differentiation between local *Bombina orientalis* populations

	Beijing	Yantai	Qingdao
Beijing		0.271***	0.772***
Yantai	0.203***		0.609***
Qingdao	0.463***	0.161***	

*R*_ST_ is based on microsatellite data (bottom left) and *Φ*_ST_ is based on mtDNA data (top right)

***p < 0.001

Despite a general lack of genetic differentiation among sampling sites in Beijing, we found a significant positive correlation between the genetic distances estimated with microsatellite data and the geographic distances among samples from ZWY and those from the other sites in Beijing (Spearman *rho* = 0.123; *p* < 0.001; Additional file 1: Fig. S2).

### Estimation of effective population sizes

Effective population size estimates (*N*_e_) at all tested minimum allele frequencies showed consistently large values (from over 1000 to infinity) for the Yantai and Qingdao populations and substantially small values for the Beijing population (*N*_e_ = 20–57, 95% CIs = 17–69) (Table 3).

**Table 3:** Recent local populations’ effective population sizes (*N*_e_) estimated using the LDNe method

Population		MAF			
		0.05	0.02	0.01	0
Beijing	*N*_e_	20.3	32.0	32.0	57.2
	95% CI	16.6–24.7	26.9–38.1	26.9–38.1	47.9–68.9
Yantai	*N*_e_	∞	1030.2	1386.7	5932.8
	95% CI	637.2–∞	439.8–∞	575.8–∞	1002.2–∞
Qingdao	*N*_e_	∞	∞	∞	∞
	95% CI	83.1–∞	139.8–∞	139.8–∞	139.8–∞

MAF, minimum allele frequency; CI, confidence interval

### Estimation of the female founder number

**Fig. 4:**
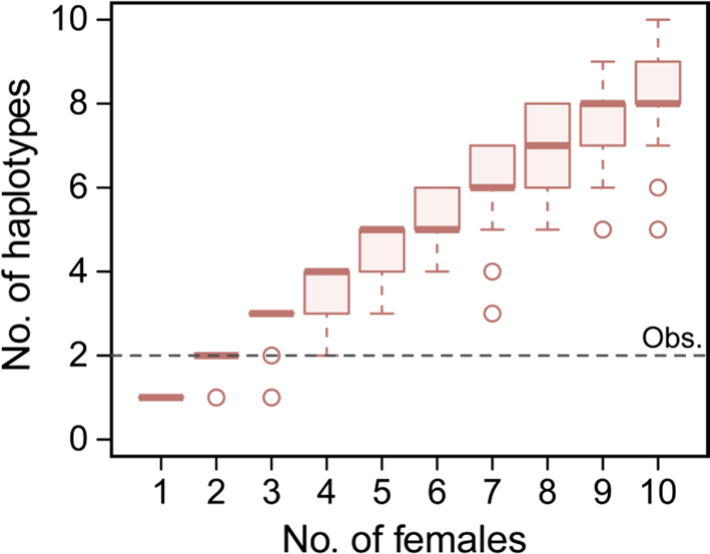
Simulation of the number of founder females needed to retain the current mtDNA diversity in the Beijing population. The central horizontal lines in the plots represent medians, the boxes indicate the first and third quartiles, the error bars represent minimum and maximum values, and the circles represent outliers. The horizontal dashed line indicates the observed number of haplotypes in the current Beijing population

We used mtDNA data with a simulation-based approach to estimate the number of female founders (i.e., the introduced females that have genetically contributed to the present population) that could retain the haplotype diversity of the Beijing population. The number of retained mtDNA haplotypes increased with an increasing number of females drawn randomly from the source population that was simulated using the observed mtDNA diversity in the Yantai population (Fig. 4). Based on this simulation, two to four founder females were most likely able to generate the observed number of haplotypes (i.e., 2) in the current Beijing population.

### Founding bottleneck and genetic drift simulations

**Fig. 5:**
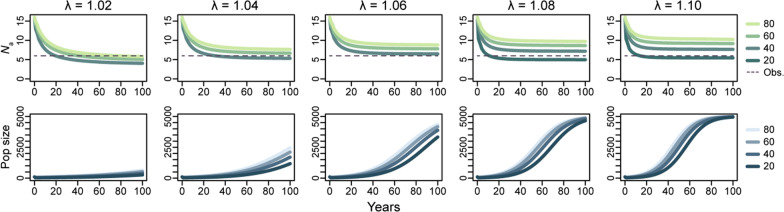
Simulation of the loss of genetic diversity over 100 years in the introduced population. The top panels show changes in the mean allele number per locus (*N*_a_) at various population growth rates (λ) when the founder population size = 20, 40, 60, or 80 individuals, and the horizontal dashed lines indicate the observed *N*_a_ in the current Beijing population (~ 90 years post introduction). The bottom panels show changes in population size with different founder sizes and λ while following a logistic growth pattern. The initial sex ratio in the founder population was set at female:male = 1:3. See Additional file 1: Fig. S3 for simulation results at 1:1, 1:6, and 1:9 female:male sex ratios

Using the microsatellite data and simulations of a population bottleneck and subsequent genetic drift under various annual population growth rate settings, we estimated founder size and post-bottleneck population demographics. Assuming the original source population possessed the same mean number of alleles per locus as the present Yantai population (*N*_a_ = 17.3; Table 1) and using the initial female:male ratio of 1:3 in the founder population (see “Methods” section), our simulations indicated that the *N*_a_ in the introduced population rapidly decreased during the bottleneck and continued to decrease because of subsequent genetic drift (Fig. 5). As the years after the introduction passed, and within 20–40 years post-introduction, the population size had gradually risen, the drift effect had attenuated, and the *N*_a_ curves had leveled off and stabilized. Notably, the larger the population growth rate (λ), the more rapidly the *N*_a_ stabilized. Population genetic diversity loss trends were similar for different founder population size settings, except that a founder size of 20 resulted in population extinction in simulations with smaller λ values (1.02–1.06). The final *N*_a_ values were higher with a larger founder size or a more rapid population growth (Fig. 5). The simulation results indicated that a λ between 1.04 and 1.08 under a logistic model was likely to generate the current population size in Beijing, while comparisons with the observed *N*_a_ suggested that the Beijing population may have expanded from a founder population of 40–60 individuals. An initial 1:1 sex ratio delivered similar population genetic trajectories, except that the population propagated even with a founder size of 20 and a small λ (Additional file 1: Fig. S3). Setting a highly male-biased initial sex ratio (i.e., female:male = 1:6 and 1:9) caused population extinction at all λ values when the founder number was fewer than 40 and 60, respectively (Additional file 1: Fig. S3).

## Discussion

### Genetic reconstruction of the introduction history

Population genetic analysis provided potential details of *B. orientalis*’s introduction in Beijing and shed light on the existence of this population’s survival in isolation for nearly a century. The Beijing population’s haplotypes nested within the Yantai haplogroup (Fig. 2), a finding that provides strong genetic evidence that the Yantai or its nearby population was the source for the Beijing population. This result also supports our sampling of the actual source population and corroborates the historical record of the translocation event [24]. Additionally, we detected strong genetic signatures of the Beijing population’s introduction, such as remarkably lower mtDNA and microsatellite diversities and distinctly different genetic clustering compared to those of the source population. The Beijing population signatures may be attributed to the combined effects of the initial severe demographic bottleneck during the introduction and the subsequent genetic drift caused by the population’s isolation and small size. Those genetic patterns also corroborate the documented introduction history that *B. orientalis* was originally translocated to Beijing in a single introduction event, and had no subsequent admixture with other populations. The individuals translocated to PKU may not have survived, as we found no *B. orientalis* during our frequent surveys of that site and adjacent areas. The PKU site and the surrounding areas, unlike the five mountainous sites where *B. orientalis* were found, are isolated from mountain habitats and located in an area of flatland, which has undergone much human interference in the last few decades. Therefore, the PKU site may not be environmentally suitable for *B. orientalis* and the individuals released there did not propagate, at least not in recent years. The present Beijing populations, which are all in foothill habitats connected by mountain ranges to the ZWY site, may have arisen entirely from the toads that were captured in Yantai and released at ZWY in 1927.

Although introduced populations typically show reduced genetic diversity compared with their source populations [12, 26], it was still striking that the mtDNA sequences of all 150 samples from Beijing possessed only two haplotypes. Considering our sample size and survey range, we believe that we have sampled most of the haplotypes present in the Beijing population. The very low haplotype diversity suggests that this population descended from a small number of introduced females. Our founder size simulations based on mtDNA data suggest that only two to four female founders were needed for the current haplotype diversity in Beijing (Fig. 4). However, this estimate assumes a complete sampling of all present haplotypes and did not account for haplotype loss following introduction. Considering that a substantial amount of genetic variation may have been lost during population establishment, and also the possibility that we may have missed rare haplotypes when sampling the Beijing population, the actual number of female founders could be considerably larger than the estimated value. Microsatellite-based simulations of the genetic effects of bottleneck and random drift suggest a male and female founder population of 40–60 toads (Fig. 5). Considering the male-biased sex ratio of the field-collected *B. orientalis* [27, 28], we assumed that the toads captured and translocated by Chengzhao Liu had a similar ratio (1:3 female to male). Thus, the founding population likely consisted of 10–15 females and 30–45 males. Overall, our results suggest that the progeny of about half of the approximately 100 toads introduced at ZWY have been propagating in Beijing for over 90 years.

A recently published study also investigated the *B. orientalis* population genetic diversity at two Beijing sites (ZWY and BWS) compared to that of the Yantai population [29]. Using a dataset of one mtDNA marker and six microsatellite loci from 63 toads, Teng et al.'s [29] results were similar to ours: lower genetic variation in the Beijing populations and strong genetic differentiation between the Beijing and Yantai populations. However, Teng et al. [29] identified five haplotypes among their Beijing samples. Sequence alignment of our and their haplotypes showed that our sequences were mostly covered by theirs, but a fragment (~ 300 bp) of their sequences was not included in our sequences. The overlapping regions of two haplotypes found in our Beijing samples are identical with those of three of their haplotypes (an additional segregation site in the non-overlapping regions further differentiated two of their haplotypes). Their remaining two haplotypes with different sites from our sequences were not detected in our study. Both of those haplotypes occurred at BWS. Further investigation is needed to determine whether those haplotypes represent local populations not uncovered by our sampling, or sequencing or assembly errors.

### Population size and range expansion

Although our estimated *N*_c_ for the Beijing population was about 1,500–4,000 individuals, recent *N*_e_ estimates, under a range of parameter settings, were merely 20–57 (95% CI: 17–69) (Table 3). Thus, the estimated *N*_e_/*N*_c_ ratio of 0.005–0.04 in the present Beijing population is a fairly low value compared to most wild populations (on average around 0.1 [30]). Although low *N*_e_ values appear to be common among amphibian species [31], the considerably smaller *N*_e_ of the Beijing population compared with those of the Yantai and Qingdao populations suggests that low *N*_e_ is not a general characteristic of the species but rather the consequence of introduction. This also indicates that the substantial demographic growth after introduction has not compensated for the low genetic diversity that resulted from founder bottlenecks and genetic drift, so the *N*_e_ has remained low in the Beijing population.

Questions about the exact distribution range of the current *B. orientalis* population in Beijing remain. The farthest Beijing location where we detected the species, YTS, was approximately 13 km from the original release site, ZWY (Fig. 1c). A similarly sized congenic species, *B. variegata*, has an individual dispersal distance up to 4 km [32]. A study of *B. orientalis* in its northeast China range suggests that both sexes are capable of long-distance (> 10 km) dispersal through continuously forested habitats [28]. Hence, we expect that the introduced *B. orientalis* population has had the ability to further expand its range, but low habitat availability and connectivity in Beijing’s highly urbanized landscape have largely contained its spread. Although more distant populations than the ones we found in our field surveys likely exist, long-distance dispersal in the species’ Beijing range may be very difficult across fragmented habitat and human-dominated landscape and thus likely rarely occur. We also found a significantly positive relationship between the genetic distances among samples and their geographic distances from the ZWY site (Additional file 1: Fig. S2), thus suggesting a radial mode of isolation-by-distance between dispersal sites and the introduction site. The population expansion front may possess lower genetic diversity due to a small number of colonizers, as well as to limited gene flow with the main population [33, 34], as shown by the low mtDNA and microsatellite diversity levels at the two Beijing sites farthest from ZWY (YTS and BDC; Fig. 1c and Table 1). Further investigation into the Beijing population size and distribution and population genetic patterns may provide insights into this introduced population’s growth and expansion rates, dispersal pattern, and habitat selectivity, as well as into the effects of population demographic and range expansions on the level and distribution of population genetic variation.

### Genetic diversity, propagule pressure, and introduction success

The relationship between population genetic diversity and introduction success has long been a topic of heated debate [35–38]. For introductions that have started with a small number of individuals, the populations exist in limited sizes for at least the initial stages of colonization. That is thus associated with significantly reduced genetic variability, increased inbreeding depression, and fixed deleterious mutations caused by demographic bottlenecks and genetic drift [39]. However, there are many examples of introduced species establishing stable populations while showing no apparent signs of inbreeding depression, despite low genetic diversity [13, 26, 40–42]. An infamous example of that is when 101 cane toads (*Rhinella marina*), a species native to South and Central America, were brought to Queensland in 1935 and, despite its low genetic diversity, their progeny spread along the Australian coasts, becoming a major invasive species in that country [43, 44]. Our study provides additional support that introduced amphibian populations may persist with low genetic variation and no gene flow from other populations. Therefore, successful establishment and persistence of introduced populations can occur without a large number of founders or multiple introductions (i.e., a large propagule pressure). Genetic, demographic, environmental, and stochastic factors can all influence the colonization process, and there does not seem to be a one-size-fits-all rule for predicting introduction outcomes [38].

Suitable ecological conditions in the recipient environment are also critical for introductions to successfully establish [45, 46]. Molecular dating using mtDNA and nuclear genes suggests that *B. orientalis* diverged from the European *Bombina* clade in the Miocene [47, 48], and the two current native ranges of *B. orientalis*, one in the Korean Peninsula and adjacent areas and the other in the Shandong Peninsula (Fig. 1a), split at 0.5–4.5 MYA [47]. It is likely that *B. orientalis*, following its divergence from the European *Bombina*, had initially occupied a larger range in East Asia, but then repeated glaciations in the Pleistocene shrank and fragmented its distribution to the extant ranges. The two peninsulas may have served as refugia for *B. orientalis* during Pleistocene glaciations when genetic exchange between the two populations was likely cut off by geographic barriers such as lowlands and the sea [47]. The successful establishment of a *B. orientalis* population in Beijing indicates that the area provides suitable habitat for the species. So, Beijing and its surrounding areas may have had suitable habitat at certain points in the past and had harbored ancient *B. orientalis* populations. Such a scenario 6000 years ago is suggested by ecological niche modeling of the historical suitable habitat ranges for this species [28].

Our case study of the introduced *B. orientalis* in Beijing offers a unique opportunity to examine the demographic and genetic dynamics of a small population that has persisted in isolation for over 30 generations. Our findings suggest that under certain circumstances biological invasions can escape the restraints of propagule pressure, and that species translocation to a non-native area (even as a conservation measure) may risk that species becoming invasive in that area. Such invasion risk may be particularly high for amphibian species with broad geographic distributions, short life cycles, frequent reproduction, and large clutch sizes—characteristics that give them superior environmental adaptability and the potential for rapid population growth and range expansions [49–52]. Therefore, great caution should be exercised to avoid accidental or intentional translocation of even a very few individuals of any foreign species. Although the European Union has assessed a low invasion potential for *B. orientalis* [53], the species has a moderate clutch size (100–300 eggs/female/year) and comparatively short life cycle [32, 54], traits that may facilitate its establishment and adaptive evolution in the introduced area. Continued monitoring of Beijing’s *B. orientalis* population will not only track its demographics and range expansion but also help us gain insights into both the founding process and the prospects for small and isolated amphibian populations.

## Conclusions

Our study provides an intriguing example of a non-native amphibian population that established from a single introduction of a small number of founders and has persisted in isolation for nearly a century. Our results corroborate the historical record of the source population for the Beijing *B. orientalis* population and demonstrate strong genetic signatures of founder bottleneck and genetic drift in that small population over time. Genetic data-based simulations suggest that the current Beijing population, which has expanded to thousands of individuals and spread at least 13 km from the initial introduction site, possesses low genetic variation and a small *N*_e_, and that the population may have descended from a limited number of toads released in 1927. The relatively high reproductive output, as well as the similar climatic and environmental conditions between their native and introduced ranges may have facilitated *B. orientalis*’ successful establishment in Beijing. Further investigation would reveal the long-term fitness effects of low genetic variability on the reproduction, adaptability, and persistence of this and other populations that experience similar genetic trajectories (such as invasive species colonizing non-native ranges), as well as aid conservation using ex situ techniques.

## Methods

### Study species, field surveys, and sampling

Adult *B. orientalis* (body length 38–45 mm) spend most of their time on land, breeding from May to July in waterbodies such as streams, ponds, ditches, and paddy fields in low-altitude (< 900 m above sea level) mountainous forests, bush lands, and swamps [55]. During the summers of 2015–2017, we surveyed water bodies at the original release sites, PKU and ZWY, and at suitable habitats within a 30 km circumscribed area surrounding those sites (Fig. 1c, Additional file 1: Table S1). We focused on low mountainous and foothill habitats, paying particular attention to mountain streams, ponds, and temporary water holes. Putative *B. orientalis* toe clips of adult toads were collected and stored in 95% ethanol at − 20 °C until DNA extraction. To compare the population genetic diversity between introduced and source populations, we collected *B. orientalis* samples from the reported source, Yantai City (Fig. 1b). With little knowledge about Chengzhao Liu’s actual collection site within the city, we chose to collect samples at Kunyushan National Nature Reserve because it is one of the best-preserved mountainous ecosystems in Yantai while most parts of the city are highly urbanized. In addition, we collected samples from the Jimo District of Qingdao City (Fig. 1b) because that area had a natural population that was allopatric with the reported source population. The Qingdao and Yantai populations were approximately 560 km apart and so were most likely genetically isolated from each other.

To estimate the population size (census size, *N*_c_) in Beijing, we conducted a one-time mark-recapture experiment in August 2015 at a waterhole at the XSP site and marked (toe-clipped) 81 toads. One week later, we returned and captured 51 toads, 21 of which were marked toads from the first visit. From that, we estimated that a total of 197 (81 × 51/21) toads visited that single waterhole during that one-week period. Based on this rough estimate, and observed population sizes at several other sampling sites, we estimated 150–200 toads visiting each site. Considering the probable existence of unidentified breeding sites (e.g., ephemeral waterholes) and based on the distribution pattern and density of the sampled sites, we estimated between 10 and 20 breeding sites in the population range within Beijing. Therefore, we expected the total *N*_c_ of the Beijing population to be about 1500–4000 individuals.

### DNA extraction and genotyping

We used the EasyPure Genomic DNA Kit (TransGen Biotech Co. LTD, Beijing, China) to extract total DNA from the collected toe clip tissues. DNA quality and concentrations were checked by agarose gel electrophoresis and a NanoDrop 2000 spectrophotometer (Thermo Fisher Scientific Inc., Waltham, MA, USA). To confirm species identity for each sample, we amplified an ~ 430-bp segment of the mtDNA 16S rRNA gene, as described in Lin et al. [56]. This sequence could discriminate *B. orientalis* from the other amphibian species in Beijing [56]. The PCR products were sequenced on an ABI PRISM 3730 Genetic Analyzer (Applied Biosystems, Foster City, CA, USA).

We analyzed the genetic diversity of two mtDNA sequences: an ~ 1071-bp fragment of the D-loop region with primers D-F1086 5’-AACCGAGTGATAACCTA-3’ and D-R2102 5’-AAATCTACAGCGTGAGT-3’ and an ~ 1170-bp fragment of the cytochrome b gene (Cytb) with primers Cytb-MNCN-GluF 5’-GAAAAACCACCGTTGTTATTCAACTACA-3’ and Cytb-Amp-P10R 5’-TTCAGYTTACAAGACYGATGCTTT-3’ [57]. PCRs were conducted in a 25-μL total volume containing approximately 2 ng tissue DNA, 10 μM of each primer, and 12.5 μL 2 × EasyTaq PCR SuperMix (TransGen Biotech). PCR conditions included an initial denaturation at 95 °C for 5 min, then 40 cycles of 95 °C for 1 min, 52 °C for D-loop or 47 °C for Cytb primers for 1 min, and 72 °C for 1 min, and a final extension at 72 °C for 10 min. The PCR products were sequenced as described above.

We also used a panel of 12 loci to generate nuclear microsatellite genotypes for all samples (Additional file 1: Table S3). These included seven microsatellite loci selected from 57 loci developed for *B*. *bombina* and *B. variegata* (40 microsatellites described in Nürnberger et al. [58]; eight in Stuckas and Tiedemann [59]; and nine in Hauswaldt et al. [60]). After testing each of the 57 loci on a subset of our samples, we retained only those seven that had adequate amplification efficiency and allele-size variability. We selected the other five loci from a panel of variable microsatellite loci that we isolated from a *B. orientalis* genomic library by using (AGAT)_8_ and (GT)_15_ biotinylated probes following protocols described in Zheng et al. [61]. Except for loci 8A, 9H, and 12F, PCRs were conducted with fluorescent-labelled forward primers under the following conditions: initial denaturation at 95 °C for 5 min, followed by 40 cycles at 95 °C for 45 s, the annealing temperature for each primer pair (Additional file 1: Table S3) for 45 s and 72 °C for 45 s, and a final extension step at 72 °C for 10 min. For loci 8A, 9H, and 12F, we followed the touchdown PCR procedure described in Hauswaldt et al. [60]. All PCR products were analyzed on an ABI 3730 XL DNA Analyzer with a GeneScan 500 LIZ Size Standard (Applied Biosystems), genotyped using GeneMarker v. 2.4.2 [62], and verified by visual examination.

### Genetic data analyses

#### Genetic diversity

Using CLUSTAL W [63], we aligned all mtDNA sequences, and deposited all unique haplotypes in GenBank (http://www.ncbi.nlm.nih.gov) with accession nos. MZ593176–MZ593237 (Additional file 1: Table S2). We estimated the number of mtDNA haplotypes and polymorphic sites, haplotype diversity (*h*), and nucleotide diversity (π) using DNASP v. 5.0 [64] for D-loop and Cytb fragments, both separately and for combined sequences. Haplotype frequencies were calculated with ARLEQUIN v. 3.11 [65, 66].

To visualize the mutational relationships and geographic distributions of mtDNA haplotypes, we constructed a haplotype network using the combined mtDNA sequences (a total length of 1827 bp) for each sample and using the median joining method implemented in NETWORK v. 5.0 [67].

For microsatellite data, allelic dropout, null allele, and scoring error occurrences were estimated in MICROCHECKER v. 2.2.3 [68], and Hardy–Weinberg equilibrium (HWE) and linkage disequilibrium (LD) between loci were evaluated in the R package GENEPOP v. 4.7.2 [69]. For multiple comparisons, *P* values were adjusted using Bonferroni corrections [70]. We used the following parameters to quantify the genetic diversity of the microsatellite loci in each population: the total number of alleles, the number of private alleles, and mean number of alleles per locus, all estimated in GENALEX v. 6.5 [71]; allelic richness and private allelic richness, calculated with rarefaction using the minimum sample size in HP-RARE [72]; the observed and expected heterozygosities, calculated using CERVUS v. 3.0.3 [73]; and the inbreeding coefficient, estimated in FSTAT v. 2.9.4 [74]. To evaluate the differences in genetic diversity between populations, we first tested data normality using the Shapiro–Wilk test and, since all tested genetic parameter values were normally distributed, we then used paired *t*-tests to compare each diversity parameter across loci between populations.

#### Population differentiation

To infer population genetic differentiation based on microsatellite data, we first used a Bayesian clustering algorithm implemented in STRUCTURE v. 2.3.4 [75]. The number of genetic clusters, *K*, was set to 1 through 7, with the largest *K* equal to the number of sampled sites (i.e., Yantai, Qingdao, and five sites in Beijing). We also ran the analysis on the Beijing samples alone with *K* set to 1 through 5. For each *K* value, we performed 10 independent runs using the admixture model with correlated allelic frequencies among clusters. Simulations were run for one million Markov chain Monte Carlo iterations with a burn-in of 100,000. The most likely *K* value was determined using the Δ*K* method [76] implemented in STRUCTURE HARVESTER [77]. The results of the 10 STRUCTURE runs for the most likely *K* value were combined using CLUMPP v. 1.1 [78] with default settings, 1,000 permutations, and the Greedy algorithm.

To assess the genetic structure among individuals, we also conducted discriminant analysis of principal components (DAPC) using the R package ADEGENET v. 2.1.3 [79]. Specifically, the microsatellite data were first subjected to a principal component analysis (PCA) and then the resulting principal components were subjected to a linear discriminant analysis (LDA) to minimize variation within groups.

To evaluate the extent of genetic differentiation among inferred genetic populations, we calculated the pairwise *R*_ST_ and *Φ*_ST_ values of our microsatellite and mtDNA data, respectively, using ARLEQUIN, and tested for genetic differentiations among the populations using GENEPOP.

Since ZWY was one of the initial sites of release and likely the founder population for the other populations we found in Beijing (see “Results”), we used Spearman’s *rho* to test the correlation between genetic and geographic distances of the samples from each site and the ZWY samples. Pairwise Nei’s genetic distance [80] between samples was estimated using microsatellite data in GENALEX. The geographic distance was calculated by the Vincenty ellipsoid distance method with function *distm* in the R package GEOSPHERE v. 1.5–14 (https://rdrr.io/cran/geosphere/).

#### Estimation of effective population sizes

Using the microsatellite data, we estimated the sampled populations’ contemporary effective population sizes (*N*_e_) by using the LD single-sample estimator in NeEstimator v. 2.1 [81]. This method, previously implemented in the program LDNe, estimates *N*_e_ based on the amount of LD created by random drift among unlinked loci and corrects for bias caused by small sample sizes [82], and the estimated *N*_e_ reflects the population size in the last few generations [83]. In a simulation-based evaluation of seven most commonly used *N*_e_ estimation methods, Gilbert and Whitlock [84] found that the LDNe method outperformed other methods, particularly in scenarios with little or no migration and when the population size was small. We obtained 95% confidence intervals (CIs) using the non-parametric jackknife option. To avoid bias driven by the presence of rare alleles in the data, we chose cutoff values of 0.00, 0.01, 0.02, and 0.05 for minimum allele frequencies. A cutoff value of 0.02 was recommended by Waples and Do [85] for sample sizes larger than 25.

#### Estimation of the female founder number

To simulate the number of female founders that would retain the observed mtDNA diversity in the Beijing population, we used an individual-based model in the R package RMETASIM v. 3.1.7 [86]. We assumed that the original source population possessed the same haplotype diversity and frequencies as the contemporary Yantai population sampled in this study. From this source population, we randomly drew 1–10 females. The number of haplotypes retained in the drawn females was obtained at the end of each simulation. We repeated the simulation 100 times for each female number and estimated the likely number of female founders by comparing the simulation results with the observed haplotype number in the current Beijing population.

#### Simulating the effects of the founding bottleneck and genetic drift

We simulated the loss of genetic diversity in the microsatellite data (measured as *N*_a_) due to the initial founding bottleneck and subsequent genetic drift over 100 years using BOTTLESIM v. 2.6 [87]. This program incorporates overlapping generations, flexible reproductive system settings, and a wide range of scenarios for population size changes. Assuming that the original source population possessed the same *N*_a_ as the contemporary Yantai population, we ran the simulation for 1000 iterations with the following parameters: life-span = 4 years, age of sexual maturation = 2 years [25, 88], completely overlapping generations, random mating, dioecious reproduction, a 1:3 female to male sex ratio in the initial introduction (based on the observed sex ratio of wild-caught *B*. *orientalis* toads [27, 28]), and a 1:1 newborn sex ratio. To assess the effect of the founder population sex ratio on subsequent population genetic diversity, we also ran simulations using more extreme founder population female to male sex ratios (1:1, 1:6, and 1:9). Our field surveys suggested that the current total *N*_c_ in Beijing was possibly in the range of a few thousand. As the species’ inhabitance and range expansion are highly dependent on suitable habitat, we excluded the possibility of an exponential growth pattern and instead used a logistic growth pattern to model population growth with the population capacity set to 5000. Liu’s record of the introduction event stated that a portion of approximately 200 toads were released at each of the two sites (ZWY and PKU), but he gave no accurate numbers [24]. Since the *B. orientalis* introduced at PKU did not seem to propagate, that site was not included in our population simulations. Assuming that a roughly even number of individuals were introduced at each site, we set a pre-bottleneck population of 100 individuals for the simulated founder population at ZWY. Those individuals’ genotypes were randomly assigned using the Yantai population genotype frequencies, and new mutations were assumed to be negligible. We tested four values for the founding population (bottleneck) size: 20, 40, 60, and 80. The post-bottleneck annual population growth rate (λ) was set to 1.02, 1.04, 1.06, 1.08, and 1.10.

## Supplementary Information

**Additional file 1: Table S1.** Site and sample size information for the *Bombina orientalis* surveyed in this study. **Table S2.** Mitochondrial DNA haplotype information. **Table S3.** Primer information for microsatellite loci. **Table S4.** Results of the Hardy–Weinberg equilibrium tests for individual microsatellite loci in each population. **Table S5.** Results of paired *t*-tests of genetic diversity parameters between the Beijing and Yantai samples. **Fig. S1.** Complete graphs of the STRUCTURE analysis of *Bombina orientalis* population genetic differentiation based on microsatellite data. **Fig. S2.** Correlation between genetic distance and geographic distance among sites in Beijing. **Fig. S3.** Simulation of the loss of genetic diversity over 100 years in the introduced Beijing population of *Bombina orientalis* at different initial sex ratios in the founder population.

**Additional file 2: Data S1.** Individual mitochondrial DNA haplotypes and microsatellite genotype data.

## Data Availability

All datasets needed to generate the results of this study are available in the online supplementary materials and in Data S1 in the paper. Unique haplotype data have been deposited in the NCBI GenBank Nucleotide Database (Accession Nos. MZ593176–MZ593237).
