# Supplementary material for: A centenary tale: population genetic insights into the introduction history of the oriental fire-bellied toad (Bombina orientalis) in Beijing
Source: BMC Ecol Evol. 2022 Oct 14;22:117. doi: 10.1186/s12862-022-02072-z (PMC9569074; doi:10.1186/s12862-022-02072-z)
Supplement: Supplementary file 1 — Additional file 1: Table S1. Site and sample size information for the Bombina orientalis surveyed in this study. Table S2. Mitochondrial DNA haplotype information. Table S3. Primer information for microsatellite loci. Table S4. Results of the Hardy–Weinberg equilibrium tests for individual microsatellite loci in each population. Table S5. Results of paired t-tests of genetic diversity parameters between the Beijing and Yantai samples. Fig. S1. Complete graphs of the STRUCTURE analysis of Bombina orientalis population genetic differentiation based on microsatellite data. Fig. S2. Correlation between genetic distance and geographic distance among sites in Beijing. Fig. S3. Simulation of the loss of genetic diversity over 100 years in the introduced Beijing population of Bombina orientalis at different initial sex ratios in the founder population. [file 12862_2022_2072_MOESM1_ESM.docx]

**Supplementery Information for:**

**A centenary tale: Population genetic insights into the introduction history of the oriental fire-bellied toad (*Bombina orientalis*) in Beijing**

Shan Zhang^1,2^, Meixi Lin^1,3^, Jiawei Liu^1,4^, Jiangce Chen^5^, Dong Liu^6^, Jindong Zhao^1,2^, Meng Yao^1,2^*

^1^School of Life Sciences, Peking University, Beijing 100871, China

^2^Institute of Ecology, College of Urban and Environmental Sciences, Peking University, Beijing 100871, China

^3^Department of Ecology and Evolutionary Biology, University of California-Los Angeles, Los Angeles, California 90095, USA

^4^Department of Ecology and Evolution, University of Chicago, Chicago, Illinois 60637, USA

^5^Mechanical Engineering Department, University of Connecticut, Storrs, Connecticut 06269, USA

^6^Department of Biology, School of Life Sciences, Southern University of Science and Technology, Shenzhen 518055, China

*Corresponding author: Meng Yao (ORCID 0000-0002-8906-1461), Email: yaom@pku.edu.cn

**Table of contents (9 pages)**

Tables S1–S5 (pages 2–7)

Figures S1–S3 (pages 8–10)

**TABLES**

**TABLE S1** **Site and sample size information for the *Bombina orientalis* surveyed in this study**

| **Site** | **Site code** | **Year** | **Lat, Long** | **Altitude (m)** | **Sample size** |
| --- | --- | --- | --- | --- | --- |
| Yantai |  | 2017 | 37.30° N, 121.70° E | 129 | 89 |
| Qingdao |  | 2015 | 39.99° N, 116.18° E | 411 | 22 |
| Beijing |  |  |  |  | 150 |
| Zhiwuyuan | ZWY | 2016 | 40.01° N, 116.21° E | 166 | (49) |
| Xiangshan Park | XSP | 2015 | 39.99° N, 116.19° E | 125 | (51) |
| Baiwangshan | BWS | 2017 | 40.03° N, 116.26° E | 164 | (22) |
| Badachu | BDC | 2017 | 39.96° N, 116.18° E | 229 | (13) |
| Yangtaishan | YTS | 2017 | 40.08° N, 116.08° E | 254 | (15) |
| Peking university | PKU | 2016 | 39.99° N, 116.30° E | 45 | — |
| Yuanmingyuan | YMY | 2016 | 40.00° N, 116.30° E | 46 | — |
| Tanzhesi | TZS | 2017 | 39.90° N, 116.02° E | 418 | — |
| Beigong Park | BGP | 2017 | 39.86° N, 116.12° E | 128 | — |
| Jingxishibatan | JXT | 2017 | 40.01° N, 115.92° E | 233 | — |
| Baiyanggou | BYG | 2017 | 40.24° N, 115.97° E | 425 | — |
| Baihujian | BHJ | 2017 | 40.12° N, 116.09° E | 170 | — |
| **Total** |  |  |  |  | 261 |

The Beijing sample size is the total of the sample sizes in parentheses.

**TABLE S2** **Mitochondrial DNA haplotype information**

| **Combined mtDNA** | | **Location** | **Cytb** | |  | **D-loop** | |
| --- | --- | --- | --- | --- | --- | --- | --- |
| Haplotype name | Haplotype combination |  | Haplotype name | Acc. No. |  | Haplotype name | Acc. No. |
| Hap01 | Cytb-1/DL-1 | BJ/YT | Cytb-1 | MZ593207 |  | DL-1 | MZ593176 |
| Hap02 | Cytb-2/DL-2 | BJ | Cytb-2 | MZ593208 |  | DL-2 | MZ593177 |
| Hap03 | Cytb-12/DL-16 | YT | Cytb-12 | MZ593218 |  | DL-16 | MZ593191 |
| Hap04 | Cytb-14/DL-20 | YT | Cytb-14 | MZ593220 |  | DL-20 | MZ593195 |
| Hap05 | Cytb-14/DL-15 | YT | Cytb-14 | MZ593220 |  | DL-15 | MZ593190 |
| Hap06 | Cytb-13/DL-15 | YT | Cytb-13 | MZ593219 |  | DL-15 | MZ593190 |
| Hap07 | Cytb-25/DL-31 | YT | Cytb-25 | MZ593231 |  | DL-31 | MZ593206 |
| Hap08 | Cytb-16/DL-19 | YT | Cytb-16 | MZ593222 |  | DL-19 | MZ593194 |
| Hap09 | Cytb-10/DL-12 | YT | Cytb-10 | MZ593216 |  | DL-12 | MZ593187 |
| Hap10 | Cytb-2/DL-25 | YT | Cytb-2 | MZ593208 |  | DL-25 | MZ593200 |
| Hap11 | Cytb-30/DL-1 | YT | Cytb-30 | MZ593236 |  | DL-1 | MZ593176 |
| Hap12 | Cytb-19/DL-11 | YT | Cytb-19 | MZ593225 |  | DL-11 | MZ593186 |
| Hap13 | Cytb-12/DL-14 | YT | Cytb-12 | MZ593218 |  | DL-14 | MZ593189 |
| Hap14 | Cytb-14/DL-17 | YT | Cytb-14 | MZ593220 |  | DL-17 | MZ593192 |
| Hap15 | Cytb-26/DL-26 | YT | Cytb-26 | MZ593232 |  | DL-26 | MZ593201 |
| Hap16 | Cytb-17/DL-11 | YT | Cytb-17 | MZ593223 |  | DL-11 | MZ593186 |
| Hap17 | Cytb-11/DL-11 | YT | Cytb-11 | MZ593217 |  | DL-11 | MZ593186 |
| Hap18 | Cytb-27/DL-21 | YT | Cytb-27 | MZ593233 |  | DL-21 | MZ593196 |
| Hap19 | Cytb-23/DL-1 | YT | Cytb-23 | MZ593229 |  | DL-1 | MZ593176 |
| Hap20 | Cytb-23/DL-11 | YT | Cytb-23 | MZ593229 |  | DL-11 | MZ593186 |
| Hap21 | Cytb-23/DL-23 | YT | Cytb-23 | MZ593229 |  | DL-23 | MZ593198 |
| Hap22 | Cytb-21/DL-23 | YT | Cytb-21 | MZ593227 |  | DL-23 | MZ593198 |
| Hap23 | Cytb-28/DL-11 | YT | Cytb-28 | MZ593234 |  | DL-11 | MZ593186 |
| Hap24 | Cytb-11/DL-24 | YT | Cytb-11 | MZ593217 |  | DL-24 | MZ593199 |
| Hap25 | Cytb-11/DL-21 | YT | Cytb-11 | MZ593217 |  | DL-21 | MZ593196 |
| Hap26 | Cytb-11/DL-13 | YT | Cytb-11 | MZ593217 |  | DL-13 | MZ593188 |
| Hap27 | Cytb-18/DL-2 | YT | Cytb-18 | MZ593224 |  | DL-2 | MZ593177 |
| Hap28 | Cytb-11/DL-2 | YT | Cytb-11 | MZ593217 |  | DL-2 | MZ593177 |
| Hap29 | Cytb-11/DL-22 | YT | Cytb-11 | MZ593217 |  | DL-22 | MZ593197 |
| Hap30 | Cytb-31/DL-21 | YT | Cytb-31 | MZ593237 |  | DL-21 | MZ593196 |
| Hap31 | Cytb-31/DL-29 | YT | Cytb-31 | MZ593237 |  | DL-29 | MZ593204 |
| Hap32 | Cytb-29/DL-21 | YT | Cytb-29 | MZ593235 |  | DL-21 | MZ593196 |
| Hap33 | Cytb-11/DL-28 | YT | Cytb-11 | MZ593217 |  | DL-28 | MZ593203 |
| Hap34 | Cytb-20/DL-22 | YT | Cytb-20 | MZ593226 |  | DL-22 | MZ593197 |
| Hap35 | Cytb-22/DL-22 | YT | Cytb-22 | MZ593228 |  | DL-22 | MZ593197 |
| Hap36 | Cytb-24/DL-22 | YT | Cytb-24 | MZ593230 |  | DL-22 | MZ593197 |
| Hap37 | Cytb-11/DL-27 | YT | Cytb-11 | MZ593217 |  | DL-27 | MZ593202 |
| Hap38 | Cytb-11/DL-30 | YT | Cytb-11 | MZ593217 |  | DL-30 | MZ593205 |
| Hap39 | Cytb-15/DL-18 | YT | Cytb-15 | MZ593221 |  | DL-18 | MZ593193 |
| Hap40 | Cytb-8/DL-4 | QD | Cytb-8 | MZ593214 |  | DL-4 | MZ593179 |
| Hap41 | Cytb-4/DL-4 | QD | Cytb-4 | MZ593210 |  | DL-4 | MZ593179 |
| Hap42 | Cytb-9/DL-10 | QD | Cytb-9 | MZ593215 |  | DL-10 | MZ593185 |
| Hap43 | Cytb-5/DL-6 | QD | Cytb-5 | MZ593211 |  | DL-6 | MZ593181 |
| Hap44 | Cytb-3/DL-5 | QD | Cytb-3 | MZ593209 |  | DL-5 | MZ593180 |
| Hap45 | Cytb-3/DL-3 | QD | Cytb-3 | MZ593209 |  | DL-3 | MZ593178 |
| Hap46 | Cytb-7/DL-8 | QD | Cytb-7 | MZ593213 |  | DL-8 | MZ593183 |
| Hap47 | Cytb-3/DL-8 | QD | Cytb-3 | MZ593209 |  | DL-8 | MZ593183 |
| Hap48 | Cytb-3/DL-9 | QD | Cytb-3 | MZ593209 |  | DL-9 | MZ593184 |
| Hap49 | Cytb-6/DL-7 | QD | Cytb-6 | MZ593212 |  | DL-7 | MZ593182 |

DL, D-loop; BJ, Beijing; YT, Yantai; QD, Qingdao; Acc. No., GenBank accession number.

**TABLE S3** **Primer information for microsatellite loci**

| **No.** | **Primer** | **Primer sequence (5'-3')** | **Repeat motif** | **Size (bp)** | **T_a_ (℃)** | **Label** | **Reference** |
| --- | --- | --- | --- | --- | --- | --- | --- |
| 1 | 8A-F | AATTTCTTAGTGCTGCCAACTTGC | (AGAT)_7_AAAGAGAT(GATA)_9_ | 326–402 | touchdown | HEX | [1] |
|  | 8A-R | GGGGAAGGGACATTTTAGCTACATAC |  |  | 60–50 |  |  |
| 2 | 9H-F | AACAGCCATTATTTAAAACCATTAG | (GATA)_9_TAAA(GATA)2GAAA(GATA)_6_ | 107–183 | touchdown | ROX | [1] |
|  | 9H-R | CAATAAAGCAGTATTTCCCAAAATG |  |  | 60–50 |  |  |
| 3 | 12F-F | ATAGGAGGTTTATAATGAAAGGGCAAC | (GATA)_9_ | 203–247 | touchdown | TAMRA | [1] |
|  | 12F-R | GATTGGATTTGGGCTATGATATTCTG |  |  | 60–50 |  |  |
| 4 | B13-F | ATATTTCTTGCTATGTTGATG | (GA)_22_ | 135–190 | 46 | TAMRA | [2] |
|  | B13-R | AATTGTTTAACTTATTTTATA |  |  |  |  |  |
| 5 | B14-F | ACTAACCTGCCACATAACTTG | (TC)_4_T(TC)_7_T (TC)_9_GC(TC)_7_ | 159–189 | 48 | HEX | [2] |
|  | B14-R | CTGGGTTTTTTAATTGGAAGG |  |  |  |  |  |
| 6 | Bv11.7-F | CAATGGTCTAAAGATTTGGCAAGG | (AC)_19_ | 160–162 | 54 | TAMRA | [3] |
|  | Bv11.7-R | ACAAAATCAAGCAGTCCCAACAAAG |  |  |  |  |  |
| 7 | Bv24.5-F | GCAATACTGAAATAAATCAAGAGGC | (CA)_8_ | 283–345 | 58 | HEX | [3] |
|  | Bv24.5-R | AGTAGGCACCTGACACTG |  |  |  |  |  |
| 8 | E136-F | CCCCTGCATCTATCTATCTATCAATC | (TCTA)_29_ | 88–154 | 62 | FAM | This study |
|  | E136-R | TAAGAGTGGCAGCAAACACG |  |  |  |  |  |
| 9 | G19-F | CGGTCTGTCTGTCTATCTGTCC | (ATCT)_9_ | 253–332 | 62 | ROX | This study |
|  | G19-R | TCAGAGGATCTACCCTTCATACAA |  |  |  |  |  |
| 10 | H102-F | CACTGGATTGATGGATA | (AC)_8_ | 170–182 | 54 | FAM | This study |
|  | H102-R | CCAATGTTTGTCTGTCT |  |  |  |  |  |
| 11 | H132-F | AGGCTGCATAACGCTCTTTG | (TAGA)_8_ | 282–346 | 58 | TAMRA | This study |
|  | H132-R | TCCATCCTTCTCTTTATCCTTCC |  |  |  |  |  |
| 12 | I165-F | TTTAGGAAGTAGACAGACACGCA | (GATA)_8_ | 221–285 | 62 | HEX | This study |
|  | I165-R | GGCTGAAAACAAACATATCCAC |  |  |  |  |  |

-F, forward primer; -R, reverse primer; T_a_, annealing temperature.

**TABLE S4 Results of the Hardy-Weinberg equilibrium tests for individual microsatellite loci** **in each population.** *F*_IS_, Weir & Cockerham's estimate [4]; **, significant deviation from HWE at *p* < 0.01 level after sequential Bonferroni correction; ***, significant deviation from HWE at *p* < 0.001 level after sequential Bonferroni correction

| **Locus** | **Yantai** | |  | **Qingdao** | |  | **Beijing** | |
| --- | --- | --- | --- | --- | --- | --- | --- | --- |
|  | ***F*_IS_** | ***p-*value** |  | ***F*_IS_** | ***p-*value** |  | ***F*_IS_** | ***p-*value** |
| 8A | 0.073 | 0.386 |  | 0.112 | 0.158 |  | 0.088 | 0.139 |
| 12F | 0.084 | 0.205 |  | −0.008 | 0.309 |  | 0.159 | 0.045 |
| E136 | 0.037 | 0.750 |  | −0.104 | 0.966 |  | 0.124 | 0.012 |
| G19 | 0.068 | 0.004** |  | 0.140 | 0.248 |  | 0.091 | 0.005** |
| H132 | −0.028 | 0.514 |  | 0.228 | 0.003** |  | 0.180 | 0.003** |
| I165 | 0.518 | 0.000*** |  | 0.866 | 0.000*** |  | 0.338 | 0.000*** |
| 9H | 0.074 | 0.037 |  | −0.072 | 0.034 |  | 0.015 | 0.960 |
| BV11.7 | 0.205 | 0.106 |  | No value | No value |  | −0.100 | 0.264 |
| Bv24.5 | 0.102 | 0.182 |  | −0.035 | 0.690 |  | 0.125 | 0.003** |
| H102 | 0.182 | 0.001*** |  | 0.296 | 0.008 |  | 0.449 | 0.000*** |
| B14 | 0.033 | 0.497 |  | 0.067 | 0.120 |  | −0.027 | 0.531 |
| B13 | 0.148 | 0.060 |  | 0.190 | 0.004** |  | 0.000 | 0.281 |

**Table S5** **Results of paired *t*-tests of genetic diversity parameters between the Beijing and Yantai samples**

| **Parameter** | ***t*-value** | ***p*-value** |
| --- | --- | --- |
| *N*_A_ | 4.6512 | 0.00070 |
| *A*_R_ | 6.6854 | 0.00003 |
| *H*_O_ | 2.6713 | 0.02174 |
| *H*_E_ | 3.4769 | 0.00518 |
| *F*_IS_ | 0.0972 | 0.9244 |

*N*_A_, total number of alleles; *A*_R_, allelic richness; *H*_O_, observed heterozygosity; *H*_E_, expected heterozygosity; *F*_IS_, inbreeding coefficient.

**FIGURES**


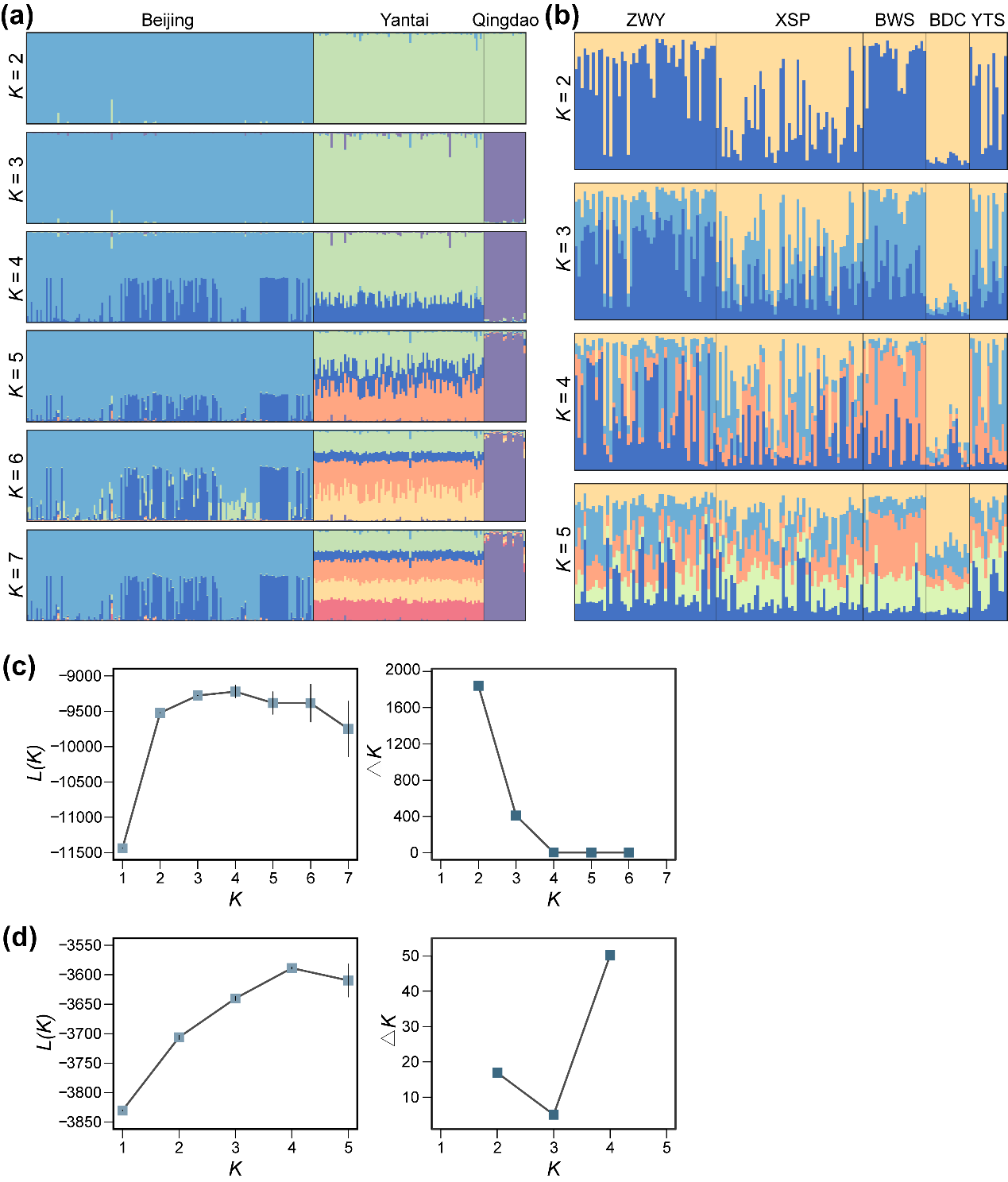


**FIG. S1** **Complete graphs of the STRUCTURE analysis of *Bombina orientalis* population genetic differentiation based on microsatellite data.** Results of the STRUCTURE analyses based on the number of genetic clusters (*K*) of (**a**) all samples and of (**b**) the Beijing samples. The corresponding means of the ln probability of *K* (left) and the rates of change (Δ*K*, right) of (**c**) all samples and (**d**) of the Beijing samples. See Fig. 1 for sampling site information.


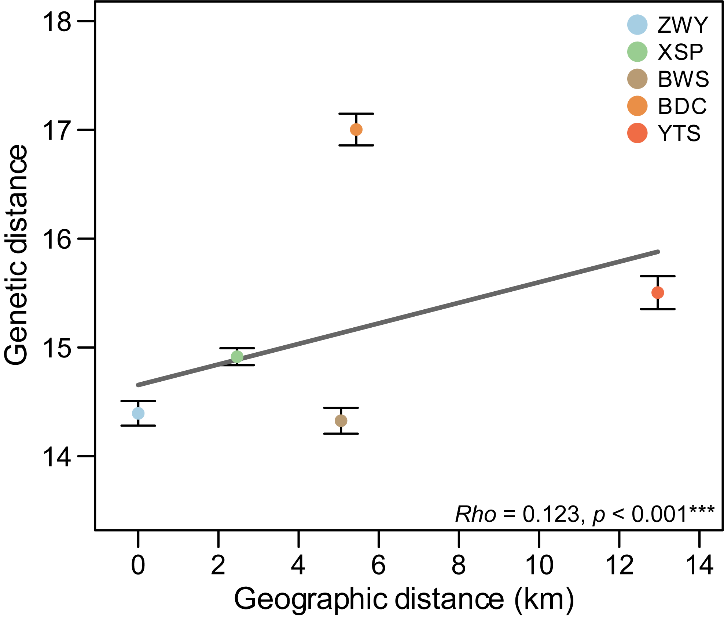


**FIG. S2** **Correlation between genetic distance and geographic distance among sites in Beijing.** The genetic distances between samples from each site and the ZWY samples were calculated using the microsatellite data. Color dots and bars represent mean (SEM), and the line represents the linear fit. See Figure 1 for sampling site information.


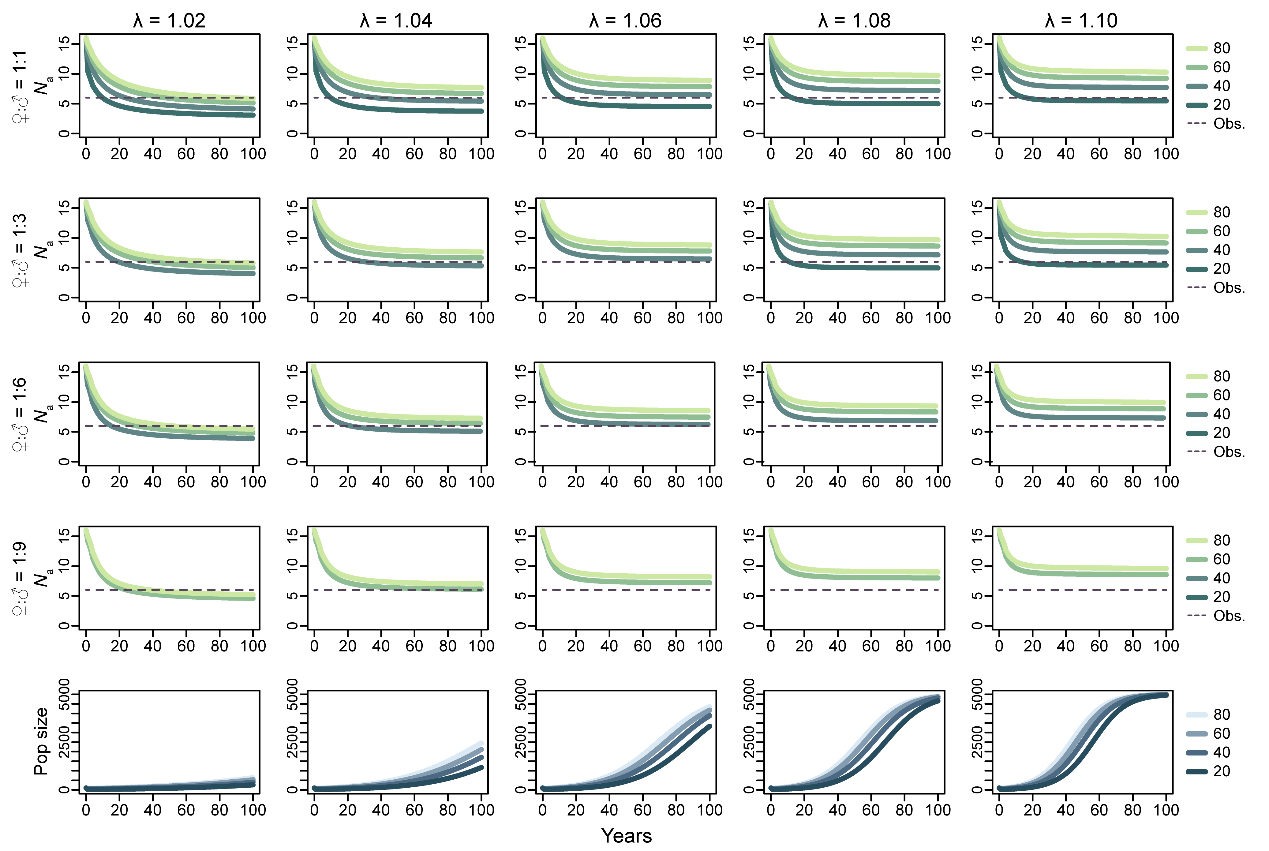


**FIG. S3** **Simulation of the loss of genetic diversity over 100 years in the introduced Beijing population of *Bombina orientalis*.** The top panels show the changes in the mean allele number per locus (*N*_a_) under different initial sex ratios in the founder population at various population growth rates (λ) when the founder population size = 20, 40, 60, and 80, and the horizontal dashed lines indicate the observed *N*_a_ in the current Beijing population (~90 years post introduction). The bottom panels show the changes in population size under different founder sizes and different λ while following a logistic growth pattern.

**References**

1. Hauswaldt JS, Schroeder C, Tiedemann R. Nine new tetranucleotide microsatellite markers for the fire-bellied toad (*Bombina bombina*). Mol Ecol Notes. 2007;7:49-52.

2. Stuckas H, Tiedemann R. Eight new microsatellite loci for the critically endangered fire-bellied toad *Bombina bombina* and their cross-species applicability among anurans. Mol Ecol Notes. 2006;6:150-52.

3. Nürnberger B, Hofman S, Förg-Brey B, Praetzel G, Maclean A, Szymura JM et al. Linkage map for the hybridising toads *Bombina bombina* and *B. variegata* (Anura: Discoglossidae). Heredity. 2003;91:136-42.

4. Weir BS, Cockerham CC. Estimating F-statistics for the analysis of population structure. Evolution. 1984;38:1358-70.
